# The positivity of estrogen receptor and progesterone receptor may not be associated with metastasis and recurrence in epithelial ovarian cancer

**DOI:** 10.1038/s41598-017-17265-6

**Published:** 2017-12-05

**Authors:** Shouzhen Chen, Xujing Dai, Yifei Gao, Fang Shen, Jingxin Ding, Qi Chen

**Affiliations:** 1 The Hospital of Obstetrics & Gynaecology, Fudan University, Shanghai, China; 2 Department of Obstetrics & Gynaecology, The University of Auckland, Auckland, New Zealand

## Abstract

The estrogen (ER) or progesterone receptors (PR) is positively associated with better clinical outcomes in ovarian cancer. Whether metastasis or recurrence of ovarian cancer is correlated with this association has not been investigated. Data on 894 women with epithelial ovarian cancer were collected and the association between ER or PR positivity and peritoneal or lymph node metastases or recurrence was analysed. ER or PR positivity was higher in high-grade, low-grade serous and endometrioid carcinoma, but lower in mucinous and clear-cell carcinoma. Significantly higher ER or PR positivity was seen in endometrioid carcinoma or high-grade serous carcinoma with peritoneal metastases, respectively, but not other subtypes. In addition, there was no significant difference in ER or PR positivity between cases with and without lymph node metastasis in these five subtypes. In recurrent high-grade serous carcinoma with peritoneal metastases (n = 103), the positivity of ER or PR was 86% and 55% respectively. Our data demonstrate that the association between ER or PR positivity and peritoneal metastases was only seen in endometrioid or high grade serous carcinoma, respectively. There was no association of ER or PR positivity and lymph node metastases. The majority of recurrent high-grade serous carcinoma with peritoneal metastases (86%) were ER positive.

## Introduction

Ovarian cancer is the third most common gynaecological malignancy and one of the leading causes of death in gynaecological cancers globally^1^. The most common type of ovarian cancer, comprising more than 90% of cases, is epithelial ovarian cancer with 4 main subtypes, including serous, mucinous, endometrioid and clear-cell carcinoma^2^.

The causes of ovarian cancer are still not fully understood, but many risk factors associated with the changes of sex hormones during a woman’s lifetime have been reported^3–8^. Estrogen, one of the main sex hormones, has been shown its effect on ovarian cell proliferation and also been suggested that women who undertake hormone replacement therapy (HRT) during menopause with estrogen for 10 years or longer increase the risk of developing ovarian cancer^9^, by the exposure of the ovarian surface epithelium to estrogen. Estrogen receptor (ER) and progesterone receptor (PR) facilitate the effects of estrogen and progesterone on proliferation and apoptosis of ovarian cancer cells^10^, but using hormone as a treatment for ovarian cancer has not yet been widely recommended. Ovarian cancer usually has a relatively poor prognosis due to late diagnosis in most cases^11^ and tumor cells have already metastasized to the peritoneal cavity at diagnosis. There is no clear clinical prognostic marker in ovarian cancer, although we have previously reported that circulating levels of Heat shock protein (HSP) 27 as well as expression of HSP27 in ovarian cancer tissue are correlated with peritoneal metastases in epithelial ovarian cancer^12,13^.

The association of ER or PR positivity and better clinical outcomes including survival rate and longevity has recently been well-documented in ovarian cancer in relation to the cancer subtypes and the subject’s ethnicities^14–21^. However, the underlying mechanisms are unknown. In addition, African American women and Asian women generally have better clinical outcomes in ovarian cancer than Caucasian women do^22,23^, but the reasons for these differences between different populations are also not clear. We recently suggested that the positivity of ER or PR in ovarian cancer is not different between Chinese women and Caucasian (revised manuscript under review), suggesting that the reason for Asian women having better clinical outcomes in ovarian cancer was not mainly because of the frequency of ER or PR positivity. This prompted us to question whether there is an association between the positivity of ER or PR and metastases and recurrence of epithelial ovarian cancer. To date such studies are limited. A recent study with a relatively small sample size (n = 16) reported higher positivity of ER and lower positivity of PR in epithelial ovarian cancer with metastases^19^. Another study with small sample size (n = 8) also reported higher positivity of ER in metastatic epithelial ovarian cancer^24^.

A better understanding of ER or PR signalling in ovarian cancer would potentially provide novel insights for improved treatment^25^. Therefore we undertook this study to investigate whether there is an association between metastases and the positivity of ER or PR in epithelial ovarian cancer, and whether the positivity of ER or PR is associated with recurrence of epithelial ovarian cancer.

## Results

### Clinical characteristics of the study cohort

The clinical and histological characteristics of study participants with primary epithelial ovarian cancer are summarised in Table 1. Of 894 cases, 568 (64%) had peritoneal metastases. Of 706 cases with data about lymph node metastases, 211 (30%) patients had lymph node metastatic disease. The median age at diagnosis in women with epithelial ovarian cancer was 53 (range 22 to 79) years. The median age at diagnosis in women with peritoneal metastases was 54 (range 22 to 79) years, which was not significantly difference from cases without peritoneal metastases (p = 0.568). The median age at diagnosis in women with lymph node metastases was 52 (range 26 to 74) years, which was not significantly difference from patients without lymph node metastases (p = 0.786).

**Table 1:** Clinical characteristics of the study population.

	Women with epithelial ovarian cancer (N = 894)
Age at diagnosis (years, median/range)	53 (22 to 79)
Histology (number, %)	
Low-grade serous carcinoma (number, %)	68 (7.6%)
High-grade serous carcinoma (number, %)	565 (63.3%)
Mucinous carcinoma (number, %)	52 (5.8%)
Endometrioid carcinoma (number, %)	62 (6.9%)
Clear-cell carcinoma (number, %)	147(16.4%)
Peritoneal metastases (number, %)	568 (63.5%)
No peritoneal metastases (number, %)	326 (36.5%)
Lymph node metastasis (number, %)	211 (30%)
No Lymph node metastasis (number, %)	495 (70%)

Data was not available on lymph node metastases in 188 cases.

### The positivity of estrogen receptor (ER) and progesterone receptor (PR) is different between epithelial ovarian cancer with and without peritoneal metastases

Due to the differnce in the nature of subtypes of epithelial ovarian cancer, we analysed the positivity of ER or PR between cases with or without peritoneal metastases according to the subtypes of epithelial ovarian cancer (Table 2). In serous carcinoma, we also divided it into low grade and high grade forms. The positivity of ERor PR in high-grade serous carcinoma was 87% (491 out of 565 cases) or 56% (316 out of 565 cases), respectively (Table 2).

**Table 2:** The association the positivity of estrogen receptor (ER) or progesterone receptor (PR) and peritoneal metastasis in epithelial ovarian cancer according to histological subtypes.

High grade serous carcinoma (n = 565)	Overall (number of cases, row %, lower, upper CL)	peritoneal metastases (n = 458)	Without peritoneal metastases (n = 107)	P value (peritoneal metastases vs without peritoneal metastases)
ER positive (number, column %)	491(87%) (83.8%, 89.57%)	399 (87%)	92 (86%)	0.751
PR positive (number, column %)	316 (56%) (51.7%, 60.1%)	248 (54%)	68 (64%)	0.001
**Low grade serous carcinoma (n = 68)**		peritoneal metastases (n = 43)	Without peritoneal metastases (n = 25)	
ER positive (number, column %)	57 (84%) (72.9%, 91.6%)	36 (84%)	21 (84%)	>0.999
PR positive (number, column %)	52 (76%) (64.6%, 85.9%)	31 (72%)	21 (84%)	0.376
Mucinous carcinoma (n = 52)*		Peritoneal metastasis (n = 13)	Without peritoneal metastasis (n = 39)	
ER positive (number, column %)	11 (21%) (11.1%, 34.7%)	1 (8%)	10 (25%)	—
PR positive (number, column %)	10 (19%) (9.6%, 32.5%)	1 (8%)	7 (18%)	—
**Endometrioid carcinoma (n = 62)**		Peritoneal metastases (n = 17)	Without peritoneal metastases (n = 45)	
ER positive (number, columne %)	52 (84%) (72.3%, 91.9%)	10 (59%)	42 (93%)	0.003
PR positive (number, columne %)	50 (81%) (68.6%, 89.6%)	11(64%)	39 (87%)	0.073
**Clear-cell carcinoma (n = 147)**		Peritoneal metastases (n = 38)	Without peritoneal metastases (n = 109)	
ER positive (number, column %)	31 (21%) (14.8%, 28.6%)	10 (26%)	21 (19%)	0.359
PR positive (number, column %)	14 (9.5%) (5.3%, 15.5%)	6 (16%)	8 (7%)	0.126

CL: confidence limits.

*Due to the sample size, statistical analysis was not conducted.

The positivity of ER or PR in low-grade serous carcinoma was 84% (57 out of 68 cases) or 76% (52 out of 68 cases), respectively. We then analysed the association of ER or PR positivity and peritoneal metastases according to the grade of serous carcinoma (Table 2). In cases of high-grade serous carcinoma (n = 565), the positivity of ER was not different between cases with and without peritoneal metastases (87% vs 86%, p = 0.751, Table 2). In contrast, the positivity of PR in cases without peritoneal metastases was significantly higher than that in cases with peritoneal metastases (54% vs 64%, p = 0.001, Table 3). In cases of low-grade serous carcinoma (n = 68), the positivity of ER or PR was not different between cases with and without peritoneal metastases (84% vs 86%, p > 0.999, or 72% vs 84%, p = 0.376, Table 3).

**Table 3:** The association the positivity of estrogen receptor (ER) or progesterone receptor (PR) and lymph node metastases in epithelial ovarian cancer according to histological subtypes.

High grade serous carcinoma (n = 425)	lymph node metastases (n = 180)	Without lymph node metastases (n = 245)	P value
ER positive (number, column %)	154 (86%)	219 (89%)	0.235
PR positive (number, column %)	108 (60%)	136 (55%)	0.372
**Low grade serous carcinoma (n = 51)**	lymph node metastases (n = 12)	Without lymph node metastases (n = 39)	
ER positive (number, column %)	11 (92%)	34 (87%)	>0.999
PR positive (number, column %)	9 (75%)	32 (82%)	0.682
Mucinous carcinoma (n = 43)*	lymph node metastasis (n = 5)	Without lymph node metastasis (n = 38)	
ER positive (number, column %)	0 (0%)	11 (29%)	—
PR positive (number, column %)	0 (0%)	10 (26%)	—
Endometrioid carcinoma (n = 52)*	lymph node metastases (n = 3)	Without lymph node metastases (n = 49)	
ER positive (number, columne %)	3 (100%)	44 (90%)	—
PR positive (number, columne %)	3 (100%)	42 (86%)	—
**Clear-cell carcinoma (n = 135)**	lymph node metastases (n = 11)	Without lymph node metastases (n = 124)	
ER positive (number, column %)	1 (9%)	26 (21%)	0.693
PR positive (number, column %)	2 (18%)	13 (11%)	0.352

*Due to the sample size, statistical analysis was not conducted.

In cases of mucinous carcinoma (n = 52), the positivity of ER or PR was 11% or 10%, respectively (Table 2). The positivity of ER or PR was 8% and 8% in case with peritoneal metastases respectively, and was 25% and 18% in cases without peritoneal metastases. Due to the sample size, statistical analysis was not conducted.

In cases of endometrioid carcinoma (n = 62), the positivity of ER or PR was 84% or 81%, respectively (Table 2). The positivity of ER in cases without peritoneal metastases was significantly higher than that in cases with peritoneal metastases (59% vs 93%, p = 0.003, Table 3). Although there was no statistical difference in the positivity of PR between cases with and without peritoneal metastases, the positivity of PR in cases with peritoneal metastases was 23% lower conpared to cases without peritoneal metastases (64% vs 87%, p = 0.073, Table 2).

In cases of clear-cell carcinoma (n = 147), the positivity of ER or PR was 21% or 9.5%, respectively (Table 2). The positivity of ER or PR was not different between cases with and without peritoneal metastases (26% vs 19%, p = 0.359 or 16% vs 7%, p = 0.126, respectively, Table 2).

We additionally compared the proportion of peritoneal metastasis between low and high grade serous carcinoma with ER or PR positivity. In cases of low grade serous carcinoma with ER positivity (n = 57), there were 36 cases (70%) with peritoneal metastasis, which was significantly lower than that in high grade serous carcinoma with ER positivity (81%, p = 0.0028). In PR positive low grade serous carcinoma (n = 52), there were 31 cases (60%) with peritoneal metastasis which was significantly lower than that in PR positive high grade serous carcinoma (78%, p = 0.005).

### The positivity of estrogen receptor (ER) and progesterone receptor (PR) is not associated with lymph node metatases in epithelial ovarian cancer

Data on lymph node metastases was available in 706 cases. We analysed the association of ER or PR positivity and lymph node metastases according to the subtypes of epithelial ovarian cancer (Table 3). In cases of high grade serous carcinoma (n = 425), the positivity of ER or PR was 86%, or 60% in cases with lymph node metastases, which was not different to the positivity of ER or PR in cases without lymph node metastases (89%, or 55%, p = 0.235, or p = 0.372, respectively). Again, in cases of low grade serous carcinoma (n = 51), the positivity of ER or PR was 92%, or 75% in cases with lymph node metastases, which was not different to the positivity of ER or PR in cases without lymph node metastases (87%, or 82%, p > 0.999, or p = 0.682, respectively).

In cases of mucinous carcinoma (n = 43, Table 3), none of the cases with lymph node metastases was ER or PR positive. While the ER or PR positivity in cases without lymph node metastases was 29% or 26%. Due to the sample size, statistical analysis was not conducted.

In cases of endometrioid carcinoma (n = 52, Table 3), all cases with lymph node metastases was ER or PR positive. In addition, the ER or PR positivity in cases without lymph node metastases was 90% or 86%. Due to the sample size, statistical analysis was also not conducted.

In cases of clear-cell carcinoma (n = 135, Table 3), the positivity of ER or PR in cases with lymph node metastases was 9% or 18%, which was not different to the positivity of ER or PR in cases without lymph node metastases (21% or 11%, p = 0.693 or 0.352, respectively).

### The positivity of estrogen receptor (ER) is proportionally higher in recurrent epithelial ovarian cancer (high-grade serous carcinoma) with peritoneal metastases

To investigate whether positivity of ER or PR is associated with recurrence of epithelial ovarian cancer, we followed up 130 recurrent epithelial ovarian cancer with peritoneal metastases (data on other 438 cases were not available). Of them, 103 (79.2%) cases were high-grade serous carcinoma. The clinical parameters of recurrent high-grade serous carcinoma are summarised in Table 4. The positivity of ER from primary tumor was 86% (89 out of 103 cases) and the positivity of PR from primary tumor was 55% (57 out of 103 cases) in recurrent high-grade serous carcinoma.

**Table 4:** Clinical characteristics of recurrent high grade serous carcinoma with peritoneal metastases.

	With recurrence (n = 103)
Age at diagnosis (years, median/range)	54 (30–79)
Time to recurrence (months, median/range)	14 (3–51)
ER positive (number, %)	89 (86%)
PR positive (number, %)	57 (55%)

## Discussion

The expression of estrogen receptor (ER) or progesterone receptor (PR) in tumor tissues has been shown to be positively correlated with the clinical outcomes in gynecological cancers including ovarian cancer, although this has been debated for many years due to study sample sizes and the influence of ethnicitic differences. The underlying mechanisms of these associations are still unknown.

High estrogen (E2) levels, one of the risk factors for ovarian cancer development were often observed in ovarian cancer patients^26^, which increases the mobility of ovarian cancer cells by the inhibition of cell to cell adhesion resulting in metastasis^27^. The ER and PR mediate the effects of estrogen or progesterone on proliferation and apoptosis of ovarian cancer cells^10^. These results suggest that positivity of ER or PR may be associated with metastasis of ovarian cancer. The most common site for recurrence of ovarian cancer is the peritoneal cavity and early peritoneal metastasis is also commonly seen in ovarian cancer^28^. Two studies with small sample sizes (less than 16) reported higher positivity of ER in peritoneal metastatic epithelial ovarian cancer^19,24^. In addition, some studies suggested higher frequency of ER positive in omental metastasis in epithelial ovarian cancer^29^, while other studies did not find any difference in the positivity of ER between omental and non-omental metastases^30^. However these studies did not divide the ovarian cancer into subtypes.

Epithelial ovarian cancer is generally divided in high grade serous, low grade serous, mucinous, endometrioid and clear-cell carcinoma. The positivity of ER or PR varies on the subtypes of epithelial ovarian cancer. In our current study, we found higher ER or PR positivity in high grade serous carcinoma, low grade serous carcinoma and endometrioid carcinoma (more than 56%, Table 2). In contrast, mucinous carcinoma and clear-cell carcinoma had lower ER or PR positivity (approximately 20%). We then analysed the association of ER or PR positivity and peritoneal metastasis. Study have reported that ER-α, one of the sub-units of ER is usually expressed in serous carcinoma including metastatic forms (review in^31^). In our current study, we surprisingly found that ER positivity was not different in high grade serous carcinoma, low grade serous carcinoma and clear-cell carcinoma between cases with and without peritoneal metastases. We also found that PR positivity was not different in low grade serous carcinoma and clear-cell carcinoma between cases with and without peritoneal metastases, whiles the PR positivity was higher in high grade serous carcinoma without peritoneal metastases. A study has suggested that PR positivity is relevant to cancer prognosis in endometrial cancer^32^. Endometrial cancer with higher positivity of PR has a good prognosis compared to that with a lower positivity of PR due to inhibition of endometrial cancer cell growth and invasion. Similarly, in ovarian cancer PR positivity is associated with improved outcome in high grade serous and endometrioid carcinoma^21^. In our current study we also found that PR positivity in high grade serous carcinoma without peritoneal metastases was significantly higher compared to high grade serous carcinoma with peritoneal metastases (p = 0.001), although the difference in PR positivity was relatively small (64% vs 54%) between these cases. This could be because of differences in the PR positivity cut-off and sample size between our current study and Sieh’s study^21^, as well as we did not have survival data available. In Sieh’s study, PR positive was defined as weak (1–50% positive) and strong (≥50% positive), while in our current study, the cut-off point of 1% positive cells was considered as ER or PR positive. Due to the sample size we were not able to further divide our study cohort into weak and strong positive groups. However our data, at least partially suggest that PR positivity is associated with reduced peritoneal metastases in high grade serous carcinoma.

Peritoneal metastasis is common in high grade serous carcinoma which shows higher ER or PR expression in ovarian cancer. In our current study we found that the positivity of ER was not different in cases with peritoneal metastases between high grade serous carcinoma and low grade serous carcinoma (87% vs 84%). In addition, the positivity of ER was also not different in cases without peritoneal metastases between high and grade serous carcinoma (86% vs 84%). This result suggests that ER positivity is not associated with the grade of serous carcinoma. Furthermore, in high grade serous carcinoma with ER or PR positive, we found that 81% or 78% cases had peritoneal metastases, which were significantly higher than that in low grade serous carcinoma with ER or PR positive (70% or 60%) (Table 4 ), but the majority of low grade serous carcinoma with ER or PR positive also had peritoneal metastases (>60%). Our result is consistent with other findings, which reported that a high proportion of low grade serous carcinoma were ER or PR positive^33,34^.

In our current study, we found significantly higher ER positivity, but not PR positivity in endometrioid carcinoma without peritoneal metastases compared to cases with peritoneal metastases (93% vs 59%), suggesting ER positivity is negatively associated with peritoneal metastases in endometrioid carcinoma. Our result is consistent with two recent studies which reported that the positivity of ER is positively associated with improved survival of endometrioid carcinoma^21,35^. It is common that the survival of cancer is better without metastasis. In our current study we also found that there was no statistical difference in the positivity of ER or PR in clear-cell carcinoma with or without peritoneal metastases. Taken together the association of ER or PR positivity and better clinical outcomes of ovarian cancer reported in the literature may be dependent on the subtypes of ovarian cancer.

Epithelial ovarian cancer may have as a primary site the epithelium of the ovary or the peritoneum itself (primary peritoneal adenocarcinoma) because the histology and clinical treatments are similar. The cancer cell metastasize onto peritoneal surfaces appears to be very direct by a physiological movement of peritoneal fluid for ovarian cancer^36,37^. Taken together our data suggest that although higher positivity of ER or PR was seen in high grade serous carcinoma and low grade serous carcinoma and endometrioid carcinoma, the positivity of ER or PR was not associated with peritoneal metastases regardless the subtypes of cancer. Our results are inconsistent with two previous studies with smaller sample sizes^19,24^, which reported higher ER or PR positivity in ovarian cancer with peritoneal metastases. The difference between our study and those studies could be because of sampling of the ovarian cancer subtypes. In those studies authors combined all the subtypes of epithelial ovarian cancer together.

Lymph node metastasis has an important effect on prognosis of epithelial ovarian cancer and clinical treatment. The association between ER or PR positivity and lymph node metastasis has not been fully investigated in ovarian cancer. In our current study, we found that the positivity of ER or PR was not statistically different between high grade serous carcinoma, low grade serous carcinoma, endometrioid carcinoma and clear-cell carcinoma with and without lymph node metastases. Lymph node metastases normally represent the predominant or only malignant tissue at the time of recurrence. Our results therefore suggest that the ER positivity may not be associated with the prognosis in epithelial ovarian cancer regardless the subtypes of cancer.

Recurrence of epithelial ovarian cancer is common, in particular in stage III or IV of cancer, which shows 70–90% or 90–95% chance of recurrence. To date the role of hormone therapy (using antiestrogens drugs, such as Tamoxifen) in recurrent epithelial ovarian cancer has not been well established. A recent review article suggested that hormone therapy may have some benefits in the treatment of recurrent ovarian cancer, if ER is positive^38^. However, the association of ER or PR positivity and recurrence of epithelial ovarian cancer has not been fully investigated. In our current study, we were only able to follow up 130 recurrent cases with peritoneal metastases. Study has reported that PR positivity is associated with improved survival in high grade serous carcinoma^21^. Of 130 recurrent cases with peritoneal metastases in our current study we followed up, 79% of cases were high grade serous carcinoma with recurrence and our data showed that 86% of cases were ER positive and 55% of cases were PR positive, not different to the proportion of all cases with peritoneal metastases. Due to the difficulty on case follow up, we do not have data available on non-recurrent high grade serous carcinoma with peritoneal metastases. We were therefore not able to compare the positivity of ER or PR between high-grade serous carcinoma with or without recurrence. However, our data showed that the majority of those recurrent cases are ER positive, suggesting frequency of ER positive is not clinically associated with the incidence of recurrence in peritoneal metastatic high-grade serous carcinoma.

The median interval to first recurrence is 18 to 24 months in ovarian cancer. However, in our current study we found that the median time to recurrence was 14 months. The difference in time to recurrence between our study and other studies may be dependent on the subtypes or the stage of cancer or the quality of treatment or ethnicity, because the clinical outcomes of ovarian cancer vary on ethnicities. It is also well-known that the status at recurrence, such as time to recurrence, site of recurrence and number of recurrent sites are associated with treatment outcomes including the sensitivity to chemotherapy.

There are some limitations to this study. The sample size of mucinous and endometrioid cancers was small. Also due to difficulties with clinical follow up, the data on non-recurrent cases were not available, so the conclusion regarding ER positivity in recurrent of ovarian cancer needs to be taken with caution and future study is required.

In summary, in our current retrospective study with large sample size, we found that the association between ER or PR positivity and peritoneal metastases was only seen in endometriod or high grade serous carcinoma, respectively. Furthermore, there was no association of ER or PR positivity and lymph node metastases regardless of subtypes. In addition, the positivity of ER is proportionately higher in recurrent epithelial ovarian cancer with peritoneal metastases. This may suggest that the positivity of ER is not a predictor for the recurrence of peritoneal metastatic epithelial ovarian cancer. The underlying mechanisms of a positive association of ER positivity and better clinical outcomes in ovarian cancer may be dependent on other factors, such as sensitivity to chemotherapy which should be investigated in future.

## Methods

This study was approved by the Ethics Committee of The Hospital of Obstetrics and Gynaecology, Fudan University, China.

### Study participants

This retrospective data was collected from the largest Obstetrics and Gynaecology university teaching hospital, The Hospital of Obstetrics and Gynaecology, Fudan University, which serves a diverse urban and rural population in China. In this study, data on 894 women with primary epithelial ovarian cancer were collected according to the electronic based medical records of patients from January 2010 to December 2015. Clinical characteristics included age at diagnosis, self-reported age at menopause, and pathological findings. Pathological findings included classification and stage of cancer, and positivity of estrogen receptor (ER) and progesterone receptor (PR) measured by immunohistochemistry.

A diagnosis of ovarian cancer was confirmed by pathological analysis of the tumor specimens from surgery. Peritoneal metastases in epithelial ovarian cancer were identified by macroscopic examination during surgery, and subsequent histological examination of the surgical specimens. Lymph node metastases were identified by histological examination of the surgical specimens. Recurrent ovarian cancer was defined based on clinical symptoms and persistently raised CA-125 levels in patients following treatment with surgery and first-line chemotherapy.

The classification of subtypes of epithelial ovarian cancer, including cancer histologic subtype and grade was determined by pathological examination of the surgical specimens using histology and with adjunctive immunohistochemistry (including WT1, TP53 and p16)^39^ (Figure 1–5 in supplementary file). According to the classification of the International Federation of Gynaecology and Obstetrics (FIGO) and World Health Organization (WHO), epithelial ovarian cancers were classified as high grade serous carcinoma, low grade serous carcinoma, mucinous carcinoma, endometrioid carcinoma and clear-cell carcinoma. The clinical and histological characteristics of epithelial ovarian cancer patients were summarized in Table 1.

### Immunohistochemistry

The expression of estrogen receptor (ER) and progesterone receptor (PR) in ovarian tissue (n = 894) was measured by immunohistochemistry on paraffin-embedded sections and read automatically (Leica Bond III, Germany) as described previously^40^. In the current study, 577 cases overlapped with previous study^40^. Briefly, antigen retrieval was performed by heating in Bond epitope retrieval solution 2 (ph 9.0) for 20 minutes. Non-specific antibody binding was blocked by incubating with Peroxide Block (Leica Microsystem) for 15 minutes. Mouse anti-human ER (1:200, Dako, clone: EP1) or PR monoclonal antibody (1:2000, Dako, clone: PgR636, Shanghai, China) were added for 1 hour at room temperature. Sections were then washed with phosphate-buffered saline (PBS, PH7.2) and incubated with biotinylated anti-mouse IgG (Dako, Shanghai, China) for 30 minutes, and after washing, sections were then incubated with streptavidin-conjugated horseradish peroxidase (Dako, Shanghai, China) for 30 minutes. The antigen–antibody complexes were incubated using 3, 3-Diaminobenzidine (DAB, Leica Microsystem) for 10 minutes and counterstained with haematoxylin (5 minutes). ER or PR positive cells were serially counted across the tumor tissue. Ovarian cancer cells with a nuclear reaction were considered as positive. The numbers of positive and negative cancer cells were counted and ER or PR positivity was represented as percentage of positive cells. The cut-off point of 1% positive cells was considered as ER or PR positive. Negative controls were performed by omitting primary antibodies.

### Statistical analysis

The number of patients with positive of estrogen or progesterone receptors was presented as a percentage with lower and upper confidence limits (95%CI), which express the lower and upper ends of the interval. The statistical difference in the number of patients with estrogen or progesterone receptor positivity in subtypes of ovarian cancer with or without metastases or recurrence was assessed by the Chi-square test or Fisher’s exact using the Prism software package (GraphPad Software Inc, San Diego, CA, USA) with *p* < 0.05 being considered as statistically significant.
